# NSAID use and unnatural deaths after cancer diagnosis: a nationwide cohort study in Sweden

**DOI:** 10.1186/s12885-021-09120-9

**Published:** 2022-01-17

**Authors:** Qing Shen, Arvid Sjölander, Erica K. Sloan, Adam K. Walker, Katja Fall, Unnur Valdimarsdottir, Pär Sparén, Karin E. Smedby, Fang Fang

**Affiliations:** 1 Department of Medical Epidemiology and Biostatistics, Karolinska Institutet, SE-171 77 Stockholm, Sweden; 2 Drug Discovery Biology Theme, Monash Institute of Pharmaceutical Sciences, Monash University, Parkville, VIC 5052 Australia; 3 Laboratory of ImmunoPsychiatry, Neuroscience Research Australia, Randwick, New South Wales 2031 Australia; 4 School of Psychiatry, University of New South Wales, Sydney, 2052 Australia; 5 Unit of Integrative Epidemiology, Institute of Environmental Medicine, Karolinska Institutet, SE-171 77 Stockholm, Sweden; 6 Clinical Epidemiology and Biostatistics, School of Medical Sciences, Örebro University, SE-701 82 Örebro, Sweden; 7 Center of Public Health Sciences, University of Iceland, IS-101 Reykjavik, Iceland; 8 Department of Epidemiology, Harvard T. H. Chan. School of Public Health, Boston, MA 02115 USA; 9 Division of Clinical Epidemiology, Department of Medicine Solna, Karolinska Institutet, SE-171 77 Stockholm, Sweden; 10 Center for Hematology, Karolinska University Hospital, SE-171 77 Stockholm, Sweden

**Keywords:** Accident, Aspirin, cancer, NSAIDs, Suicide, Cohort study

## Abstract

**Background:**

Cancer patients experience increased risk of death from accident and suicide. Cognitive impairment induced by cancer-related inflammation and stress-related psychiatric symptoms may be underlying mechanisms. We therefore studied the association between use of nonsteroidal anti-inflammatory drugs (NSAIDs) and risk of these outcomes.

**Methods:**

Following a cohort of 388,443 cancer patients diagnosed between October 2005 and December 2014 in Sweden, we ascertained dispense of aspirin or non-aspirin NSAIDs from 3 months before cancer diagnosis onward and defined the on-medication period as from date of drug dispense until the prescribed dosage was consumed. Follow-up time outside medicated periods and time from unexposed patients were defined as off-medication periods. We used Cox models to estimate hazard ratios (HRs) of death due to suicide or accident, by comparing the on-medication periods with off-medication periods.

**Results:**

In total, 29.7% of the cancer patients had low-dose aspirin dispensed and 29.1% had non-aspirin NSAIDs dispensed. Patients with aspirin use were more likely to be male than patients without aspirin use. Compared with off-medication periods, there was a 22% lower risk of accidental death (*N* = 651; HR 0.78, 95% confidence interval [CI]: 0.70 to 0.87) during on-medication periods with aspirin. The use of aspirin was not associated with risk of suicide (*N* = 59; HR 0.96, 95% CI: 0.66 to 1.39). No association was noted between use of non-aspirin NSAIDs and the risk of suicide (*N* = 13; HR 0.95, 95% CI: 0.42 to 2.18) or accidental death (N = 59; HR 0.92, 95% CI: 0.68 to 1.26).

**Conclusions:**

Intake of low-dose aspirin after cancer diagnosis was associated with a lower risk of unnatural deaths among cancer patients.

**Supplementary Information:**

The online version contains supplementary material available at 10.1186/s12885-021-09120-9.

## Introduction

The impact of cancer and cancer treatment on cognition and mood changes has been well-characterized [1]. While improved treatment has increased the number of cancer survivors, studies have demonstrated that cancer patients experience increased symptoms of cognitive impairment, anxiety, depression, and fatigue during and after cancer treatment [2]. These symptoms subvert normal functioning of the central and peripheral nervous system, which may manifest in pathophysiological process of health consequences [3]. As a result, more cancer patients and survivors now suffer from stress-related health outcomes, including fatal and non-fatal injuries, accident, and suicide [4–7].

.It is plausible that biological mechanisms such as inflammation induced by cancer or cancer treatment contribute to increased risk of accidents and other unnatural deaths in cancer patients. Inflammation is a hallmark of cancer and increases in response to some cancer treatments such as chemotherapy [8]. Several studies have identified a relationship between circulating pro-inflammatory markers and symptoms of cognitive impairment, mood and stress-related disorders, as well as peripheral neuropathy both at cancer diagnosis and during or soon after cancer treatment [9, 10]. Markers of inflammation such as increased neutrophil-to-lymphocyte ratios were noted up to 20 years after cancer treatment and coincide with symptoms of cognitive decline [11]. In a recent study, we demonstrated that aspirin was able to block tumour-induced memory impairment in mouse models of breast cancer, suggesting its potential to combat cancer-related cognitive and mood symptoms [12]. In humans, aspirin and non-aspirin NSAID use was shown to be associated with decreased risk of depression and depressive symptoms [13], especially in terms of continued use of low-dose aspirin [14].

.Taking advantage of the Swedish Prescribed Drug Register and a national cohort of incident cancer patients in Sweden, we aimed to explore the association between use of low-dose aspirin and non-aspirin NSAIDs and the risk of suicide or death due to accident following a cancer diagnosis.

## Methods

### Study design

Due to the availability of data on medication since July 2005, we identified all patients with a newly diagnosed cancer (*N* = 403,322) between October 2005 and December 2014 from the Swedish Cancer Register, which includes almost complete information on all cancers diagnosed in Sweden since 1958 onward [15]. All patients were cross-linked to the Swedish Causes of Death and Migration Registers using the personal identification numbers assigned uniquely to all residents in Sweden [16]. We excluded 155 patients who died and 14,724 patients who emigrated before cancer diagnosis, leaving 388,443 patients to be followed from the date of cancer diagnosis, until emigration (Migration Register), death (Causes of Death Register), or December 31, 2014, whichever occurred first. We used 7th Swedish revision of the International Classifications of Diseases (ICD) codes to classify different cancer types (Supplementary Materials, Table S1).

### Ascertainment of exposures

The Swedish Prescribed Drug Register contains information on prescribed and dispensed medications from all Swedish pharmacies since July 2005 [17]. All pharmacies, retailers and wholesalers across the country are obligated to report the sales on monthly basis with overall very good data quality [17]. Unused drugs are advised to be returned to the pharmacies for incineration. The proportion of all returned drugs was around 2.3–4.6% of the dispensed volume [18]. Medications in this register are classified according to the Anatomical Therapeutic Chemical (ATC) system [19]. The register includes information on medicine types, prescription and dispensing dates, quantity, defined daily dose and prescription text [17]. Low-dose aspirin and most NSAIDs cannot be purchased over-the-counter without a prescription in Sweden [20]. Patients who had medications dispensed with ATC codes B01AC06, B01AC30 and B01AC56 were considered as medicated with lose-dose aspirin (limited to daily dose of 75 or 160 mg). We focused on low-dose aspirin that tends to be used in long term in the present study, because of its potential effect of reducing stress-related outcomes [14, 21]. Patients who had medications dispensed with ATC code M01A were considered as medicated with non-aspirin NSAIDs.

We identified all low-dose aspirin and non-aspirin NSAIDs dispensed from 3 months before cancer diagnosis until the end of follow-up, because prescription drugs are dispensed for up to a three-month supply in Sweden. Multiple prescriptions at the same dispense date for the same medicine (2% for aspirin, 1.6% for non-aspirin NSAIDs) were summed up and unused medicines returned to the pharmacies were extracted from the amount of the previous dispense (0.3% for aspirin, 0.3% for other NSAIDs). Because of the time-varying nature of medication use, we constructed on- and off-medication periods for each patient after cancer diagnosis through information on dispense date and dosage according to the prescription text. The on-medication period was defined as the interval from the most recent dispense date of a specific NSAID, until the last day when the dispensed drug was estimated to be consumed. Time periods outside the on-medication periods among patients that had ever used NSAIDs, and among patients that did not use any NSAID during follow-up, were defined as off-medication periods (Supplementary Materials Fig. S1).

Because the defined daily dose does not necessarily correspond to the recommended or prescribed daily dose, we estimated days on medication as the division of the total amount of dispensed drug by the prescribed daily dosage per medicated period for each patient. The information on prescribed daily dosage for each on-medication period was extracted from the prescription text, or from the defined daily dose when the prescription text was not available (25% for aspirin, 4% for non-aspirin NSAIDs).

### Ascertainment of completed suicide and death due to accident

The Swedish Causes of Death Register collects nationwide information from 1961 onward, including dates and the underlying and contributing causes of death [22], with high accuracy [23]. We used the 10th Swedish revision of ICD (International Classification of Diseases) codes X60-X84, V01-X59, and Y85-Y86 to ascertain deaths from suicide and accident (Supplementary Materials Table S2). Death due to accident was further classified as deaths due to transport accident, fall, accidental threat to breathing, unspecified fracture, or others.

### Covariables

Patients’ use of drug as well as their general health status may confound the association between aspirin and non-aspirin NSAIDs and risk of unnatural deaths from suicide and accident. To control for these confounding factors, we included socioeconomic status (education, occupation, cohabitation status), mental health status (history of psychiatric disorder), and general health status (Chronic Disease Score) as covariates. The Longitudinal Integration Database for Health Insurance and Labour Market Studies (LISA) was established by Statistics Sweden and collects data from labor market, and educational and social sectors annually for individuals over 16 years of age [24]. Information on the highest educational level, occupation, and cohabitation status at the time of diagnosis was retrieved from LISA for all cancer patients. Chronic Disease Score is a measure of comorbidity based on the aggregated number of prescribed medications. The score is a summary of weights from each comorbidity category represented by medication classes [25, 26]. As a proxy of general health status, we calculated a Chronic Disease Score, based on the prescribed medications before cancer diagnosis, for each patient [25], after excluding anxiolytics, antidepressant, antipsychotic, and anti-inflammatory medications because of their close relationship with the exposure or other covariables. We defined history of psychiatric disorders as having any inpatient or outpatient hospital visit for psychiatric disorders from 1987 onward using the ICD-9 codes 290–319 and ICD-10 codes F10-F99. Because of the close link between psychiatric disorders and suicide and death due to accident [27, 28], we updated this variable per on- or off-medication period for each patient.

### Statistical analysis

We first described the demographic and clinical characteristics of the cancer patients, with and without medications, including sex, age at cancer diagnosis, calendar period of diagnosis (2005–2008, 2009–2011 or 2012–2014), highest educational level (> 12 years as after secondary school, 9–12 years as secondary school, < 9 years as primary school, or missing), occupation (blue collar, white collar, farmers, self-employed, retired or unemployed, or unclassified or missing), cohabitation status (cohabitation, non-cohabitation, or missing), cancer type (prostate cancer, breast cancer, colorectal cancer, non-melanoma skin cancer, hematopoietic malignancy, lung cancer, severe cancer [esophagus, liver and pancreas] and others), cancer stage (localized limited, localized advanced, regional spread, distant metastasis, unknown, or not applicable), history of psychiatric disorders (no or yes), and Chronic Disease Score (0, 1–2, 3–5, or > 6).

To assess the impact of taking aspirin and non-aspirin NSAIDs separately, we used Cox proportional hazards regression models to estimate the hazard ratios (HRs) and 95% confidence intervals (CIs) of suicide and death due to accident after cancer diagnosis, by comparing the on-medication periods with the off-medication periods of aspirin, and of non-aspirin NSAIDs, separately. In the first model, we used time since cancer diagnosis as the underlying timescale, and additionally adjusted the estimates for age at diagnosis (continuous variable), sex, highest educational level, occupation, cohabitation status, and calendar year of diagnosis. A continuous function of time was modeled with restricted cubic splines. In a second model, we additionally adjusted for cancer type, cancer stage, and Chronic Disease score (continuous variable). In a third model, in addition to all variables adjusted for the second model, we also adjusted for history of psychiatric disorders. The three models were designed to demonstrate the roles of different covariables on the studied associations. To illustrate the temporal pattern of the association, we also estimated the hazard as a function of time using restricted cubic splines with three knots that were evenly distributed along time at risk, based on the third model.

We performed sensitivity analyses. First, to assess the validity of our definition for on- and off-medication period, we defined the first month after each on-medication period also as on-medication period rather than off-medication period. Second, to separately evaluate the association for first-time use and repeated use of the medications, for each on- and off-medication period, we defined as with previous use if there was a previous on-mediation period since July 2005, and as without previous use if there was no previous on-medication period. Third, because a proportion of cancer patients had unknown stage at diagnosis, in a sensitivity analysis, we imputed unknown cancer stage to assess the influence of such missingness on the main results. Fourth, to exclude potential influence from other NSAIDs when investigating the effect of aspirin use (and vice versa), we compared the on-medication periods of aspirin or non-aspirin NSAIDs with off-medication periods with neither aspirin nor non-aspirin NSAIDs. Finally, to assess the role of other medications with potential impact on cognitive function and psychiatric symptoms, we performed additional analysis with further adjustment for use of opioids (ATC code N02A), use of anxiolytics (ATC code N05B), or use of antidepressants (ATC code N06A).

Because an association was mainly noted between the use of low-dose aspirin and accidental death, we also performed several secondary analyses to assess the robustness of this finding. First, because patients are often asked to discontinue aspirin use during surgical treatment to avoid major bleeding [29], we performed additional analysis to separately assess the studied associations within first year (as a proxy for the time window of primary cancer treatment including surgery) and beyond first year after cancer diagnosis. Second, to further alleviate the concern of residual confounding, we separately compared the on-medication periods with off-medication periods of the same individuals (within-individual comparison). Third, because individuals with different characteristics have been shown to have different risk of accidental death following a cancer diagnosis [30, 31], we separately analyzed the association by sex, age, cancer type, cancer stage, history of psychiatric disorders, Chronic Disease Score, highest educational level, cohabitation status, and employment status (employed vs. retired or unemployed). Finally, in addition to any death due to accident, we separately studied the association by major causes of accidental death.

We found no major violation of the proportional hazards assumption in all analyses by plotting Schoenfeld residuals. All analyses were performed in SAS 9.4 (SAS Institute) and STATA 14.1 (StataCorp LP, College Station, USA).

The study was approved by the Regional Ethical Review Board in Stockholm, Sweden (Dnr 2015/1574–31). Individual informed consent was waived in this approval.

## Results

In total, 29.7% of the cancer patients had low-dose aspirin dispensed and 29.1% had non-aspirin NSAIDs dispensed. Compared with patients without aspirin use, patients with aspirin use were more likely to be male, older at cancer diagnosis, less educated, unemployed or retired, and have higher Chronic Disease Score (Table 1). Patients with non-aspirin NSAID use were younger, more educated, more likely to be cohabitating, and had lower Chronic Disease Score, compared with patients without use of non-aspirin NSAIDs. A higher incidence of death due to accident than suicide was consistently noted during the follow-up (Supplementary Materials Fig. S2).

**Table 1:** Baseline characteristics of cancer patients according to their use of low-dose aspirin and non-aspirin NSAIDs from 3 months before diagnosis onward, a cohort study of 388,443 cancer patients diagnosed between Oct 2005 and Dec 2014 in Sweden

	Low-dose aspirin		Non-aspirin NSAIDs		Patients without use of either medication (*N* = 192,333)
	Patients without use of low-dose aspirin (*N* = 273,123)	Patients with use of low-dose aspirin (*N* = 115,320)	Patients without use of non-aspirin NSAIDs (*N* = 275,449)	Patients with use of non-aspirin NSAIDs (*N* = 112,994)	
Male, N (%)	134,055 (49.1)	70,209 (60.9)	145,684 (52.9)	58,580 (51.8)	95,413 (49.6)
Mean age at diagnosis (SD), year	65.27 (13.0)	73.70 (10.2)	69.18 (12.8)	64.35 (12.2)	66.70 (13.0)
Calendar year of diagnosis, N (%)					
2005–2008	80,797 (29.6)	43,430 (37.6)	71,183 (25.8)	53,044 (46.9)	45,283 (23.5)
2009–2011	91,471 (33.5)	40,789 (35.4)	92,512 (33.6)	39,748 (35.2)	62,482 (32.5)
2012–2014	100,855 (36.9)	31,101 (27.0)	111,754 (40.6)	20,202 (17.9)	84,568 (44.0)
Highest educational level, N (%)					
Beyond secondary school	71,038 (26.0)	19,899 (17.3)	62,018 (22.5)	28,919 (25.6)	48,441 (25.2)
Secondary school	111,169 (40.7)	41,880 (36.3)	106,489 (38.7)	46,560 (41.2)	76,880 (40.0)
Primary school	88,207 (32.3)	51,940 (45.0)	103,726 (37.6)	36,421 (32.2)	64,993 (33.8)
Missing	2709 (1.0)	1601 (1.4)	3216 (1.2)	1094 (1.0)	2019 (1.0)
Occupation, N (%)					
Blue collar	23,767 (8.7)	3498 (3.0)	16,918 (6.1)	10,347 (9.2)	14,852 (7.7)
White collar	52,211 (19.1)	7721 (6.7)	37,922 (13.8)	22,010 (19.5)	33,300 (17.3)
Farmers	2079 (0.8)	625 (0.5)	1873 (0.7)	831 (0.7)	1452 (0.8)
Self-employed	9401 (3.4)	2420 (2.1)	7674 (2.8)	4147 (3.7)	6117 (3.2)
Retired or unemployed	143,688 (52.6)	86,694 (75.2)	166,437 (60.4)	63,945 (56.6)	103,998 (54.1)
Unclassified or missing	41,946 (15.4)	14,355 (12.5)	44,597 (16.2)	11,704 (10.4)	32,614 (16.9)
Cohabitation status, N (%)					
Cohabitating	136,088 (49.8)	56,219 (48.8)	129,433 (47.0)	62,874 (55.6)	91,427 (47.5)
Non-cohabitating	106,358 (38.9)	46,411 (40.3)	107,994 (39.2)	44,775 (39.6)	74,280 (38.6)
Missing	30,677 (11.2)	12,690 (11.0)	38,022 (13.8)	5345 (4.7)	26,626 (13.8)
Cancer type, N (%)					
Prostate cancer	49,566 (18.1)	26,095 (22.6)	49,439 (18.0)	26,222 (23.2)	32,590 (16.9)
Breast cancer	43,544(15.9)	10,755 (9.3)	36,298 (13.2)	18,001 (15.9)	28,985 (15.1)
Colorectal cancer	30,024 (11.0)	13,221 (11.5)	31,576 (11.5)	11,669 (10.3)	21,764 (11.3)
Non-melanoma skin cancer	14,972 (5.5)	10,289 (8.9)	20,044 (7.3)	5217 (4.6)	11.894 (6.2)
Hematopoietic malignancy	18,518 (6.8)	9425 (8.2)	21,340 (7.7)	6603 (5.8)	14.155 (7.4)
Lung cancer	18,017 (6.6)	8693 (7.5)	19,965 (7.2)	6745 (6.0)	13,210 (6.9)
Severe cancer (esophagus, liver and pancreas)	12,267 (4.5)	4494 (3.9)	13,829 (5.0)	2932 (2.6)	10,055 (5.2)
Others	86,215 (31.6)	32,348 (28.1)	82,917 (30.1)	35,646 (31.5)	59,680 (31.0)
Cancer stage at diagnosis^a^, N (%)					
Localized limited	79,238 (29.0)	28,324 (24.5)	75,534 (27.4)	32,028 (28.3)	55,302 (28.7)
Localized advanced	16,403 (6.0)	7570 (6.6)	16,778 (6.1)	7195 (6.4)	11,295 (5.9)
Regional spread	29,086 (10.7)	10,704 (9.3)	26,872 (9.8)	12,918 (11.4)	19,221 (10.0)
Distant metastasis	29,759 (10.9)	10,668 (9.2)	30,902 (11.2)	9525 (8.4)	22,431 (11.7)
Unknown	100,119 (36.7)	48,629 (42.2)	104,015 (37.8)	44,733 (39.6)	69,929 (36.4)
Not applicable	18,518 (6.8)	9425 (8.2)	21,348 (7.7)	6595 (5.8)	14,155 (7.3)
History of psychiatric disorders, N (%)					
No	239,491 (87.7)	99,110 (85.9)	239,975 (87.1)	97,777 (86.5)	167,971 (87.3)
Yes	33,632 (12.3)	16,210 (14.1)	35,474 (12.9)	15,217 (13.5)	24,362 (12.7)
Chronic Disease Score, N (%)					
0	105,699 (38.7)	9004 (7.8)	75,296 (27.3)	39,407 (34.9)	70,037 (36.4)
1–2	111,484 (40.8)	28,884 (25.1)	97,093 (35.3)	43,275 (38.3)	77,666 (40.4)
3–5	49,854 (18.3)	65,309 (56.6)	88,137 (32.0)	27,026 (23.9)	39,307 (20.4)
> 6	6086 (2.2)	12,123 (10.5)	14,923 (5.4)	3286 (2.9)	5323 (2.8)

Abbreviations: NSAID, non-steroidal anti-inflammatory drugs

^a^Defined by European Network of Cancer Registries Condensed TNM Scheme and staging system: localized limited (T-localized/N0/M0 or FIGO 0-I), localized advanced (T-advanced/N0/M0 or FIGO II), regional spread (any T/N+/M0 or FIGO III), distant metastasis (any T/any N/M+ or FIGO IV), or unknown stage. Hematological malignancies were classified as not applicable

### Completed suicide

**Fig. 1:**
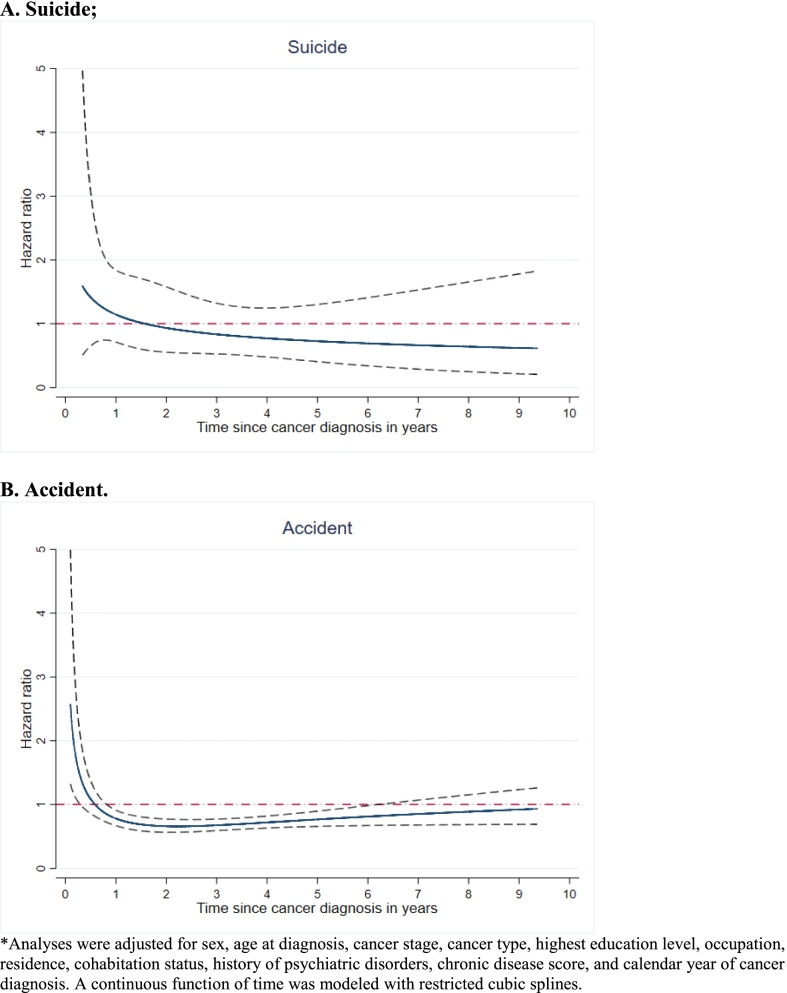
Association of low-dose aspirin use with risk of death due to suicide or accident after cancer diagnosis by time since cancer diagnosis, a cohort study of 388,443 cancer patients diagnosed between Oct 2005 and Dec 2014 in Sweden*. **A**. Suicide; **B**. Accident. *Analyses were adjusted for sex, age at diagnosis, cancer stage, cancer type, highest education level, occupation, residence, cohabitation status, history of psychiatric disorders, chronic disease score, and calendar year of cancer diagnosis. A continuous function of time was modeled with restricted cubic splines

There were 287 completed suicides observed during follow-up, among which 59 occurred during on-medication period of low-dose aspirin, whereas 13 occurred during on-medication period of non-aspirin NSAIDs (Table 2). Neither aspirin use (HR 0.96, 95% CI: 0.66 to 1.39) nor use of non-aspirin NSAIDs (HR 0.95, 95% CI: 0.42 to 2.18) was associated with a lower risk of suicide. There was no clear variation of the null association by time since cancer diagnosis (Fig. 1A).

**Table 2:** Association of NSAID use with risk of death due to suicide or accident after cancer diagnosis, a cohort study of 388,443 cancer patients diagnosed between Oct 2005 and Dec 2014 in Sweden

Characteristics	Completed suicide			Death due to accident		
	N	Crude IR (per 1000 person-years)	HR (95% CI)	N	Crude IR (per 1000 person-years)	HR (95% CI)
**Low-dose aspirin**						
Off medication (Ref) ^a^	228	0.23	1.0	1627	1.63	1.0
On medication ^b^	59	0.25	0.92 (0.64–1.31)	651	2.80	0.88 (0.80–0.98)
As above + cancer stage, cancer type and Chronic Disease Score	–	–	1.01 (0.69–1.47)	–	–	0.79 (0.71–0.88)
As above + history of psychiatric disorders	–	–	0.96 (0.66–1.39)	–	–	0.78 (0.70–0.87)
**Non-aspirin NSAIDs**						
Off medication (Ref) ^a^	274	0.02	1.0	2219	0.15	1.0
On medication ^b^	13	0.03	1.02 (0.45–2.32)	59	0.13	0.96 (0.71–1.30)
As above + cancer stage, cancer type and Chronic Disease Score	–	–	1.01 (0.44–2.32)	–	–	0.92 (0.68–1.25)
As above + history of psychiatric disorders	–	–	0.95 (0.42–2.18)	–	–	0.92 (0.68–1.25)

Abbreviations: NSAID, non-steroidal anti-inflammatory drugs; IR, incidence rate; HR, hazard ratio; CI, confidence interval

^a^Off medication time included follow-up time accumulated among patients without any dispensed NSAIDs during follow-up, as well as the non-medicated periods from patients that had any dispensed NSAIDs

^b^Analyses were adjusted for sex, age at diagnosis, calendar year of cancer diagnosis, highest educational level, occupation and cohabitation status; time since cancer diagnosis was used as the underlying timescale

The results did not change when including 1 month after on-medication period as exposed period (Supplementary Materials Table S3), when comparing to periods without prescription of any NSAIDs (Supplementary Materials Table S4), or with further adjustment for use of other medications (Supplementary Materials Table S5). No statistically significant association was noted for the risk of suicide in relation to either first-time or repeated use of either medication (Table 3). Imputation of unknown cancer stage rendered also similar results (Supplementary Materials Table S6).

**Table 3:** Association of NSAID use with risk of death due to suicide or accident after cancer diagnosis, separate analysis by status of previous medication use, a cohort study of 388,443 cancer patients diagnosed between Oct 2005 and Dec 2014 in Sweden

Characteristics	Completed suicide			Death due to accident		
	N	Crude IR (per 1000 person-years)	HR (95% CI)	N	Crude IR (per 1000 person-years)	HR (95% CI)
**Low-dose aspirin**						
**Without previous use**						
Off medication (Ref) ^a^	187	0.22	1.0	1060	1.22	1.0
On medication (first-time use) ^b^	10	0.53	1.83 (0.84–3.96)	60	3.20	1.36 (1.03–1.80)
**With previous use**						
Off medication (Ref) ^a^	41	0.30	1.0	567	4.20	1.0
On medication (repeated use) ^b^	49	0.23	0.69 (0.42–1.13)	591	2.77	0.58 (0.51–0.66)
	*P for interaction < 0.001*			*P for interaction < 0.001*		
**Non-aspirin NSAIDs**						
**Without previous use**						
Off medication (Ref) ^a^	128	0.25	1.0	1233	2.38	1.0
On medication (first-time use) ^b^	3	0.46	0.91 (0.13–6.55)	19	2.94	1.81 (1.10–2.96)
**With previous use**						
Off medication (Ref) ^a^	146	0.21	1.0	986	1.45	1.0
On medication (repeated use) ^b^	10	0.33	0.92 (0.37–2.29)	40	1.30	0.77 (0.52–1.13)
	*P for interaction = 0.99*			*P for interaction = 0.008*		

Abbreviations: NSAID, non-steroidal anti-inflammatory drugs; IR, incidence rate; HR, hazard ratio; CI, confidence interval

^a^ Off medication time included follow-up time accumulated among patients without any dispensed NSAIDs during follow-up, as well as the non-medicated periods from patients that had any dispensed NSAIDs

^b^ Analyses were adjusted for sex, age at diagnosis, calendar year of cancer diagnosis, highest educational level, occupation, cohabitation status, cancer stage, cancer type, Chronic Disease Score and history of psychiatric disorders; time since cancer diagnosis was used as the underlying timescale

### Death due to accident

There were in total 2278 deaths due to accident during follow-up, among which 651 occurred during on-medication periods of low-dose aspirin, whereas 59 occurred during on-medication periods of non-aspirin NSAIDs (Table 2). Aspirin use was associated with a 22% lower risk of death due to accident (HR 0.78, 95% CI: 0.70 to 0.87). The risk reduction in relation to aspirin use was also noted when comparing medicated periods to off-medicated periods of the same patient (HR 0.87, 95% CI: 0.77 to 0.98). No association was however noted for non-aspirin NSAIDs (HR 0.92, 95% CI: 0.68 to 1.26). The risk reduction in death due to accident, in relation to use of low-dose aspirin was mainly noted from 1 year until 6 years after cancer diagnosis (Fig. 1B).

The results did not change when including 1 month after on-medication period as exposed period (Supplementary Materials Table S3), when comparing to periods without prescription of any NSAIDs (Supplementary Materials Table S4), or with additional adjustment for use of other medications (Supplementary Materials Table S5). We found a lower risk of death due to accident in relation to repeated use of low-dose aspirin (HR 0.58, 95% CI: 0.51 to 0.66) but not first-time use (Table 3). Imputation on unknown stage rendered similar results (Supplementary Materials Table S6).

**Fig. 2:**
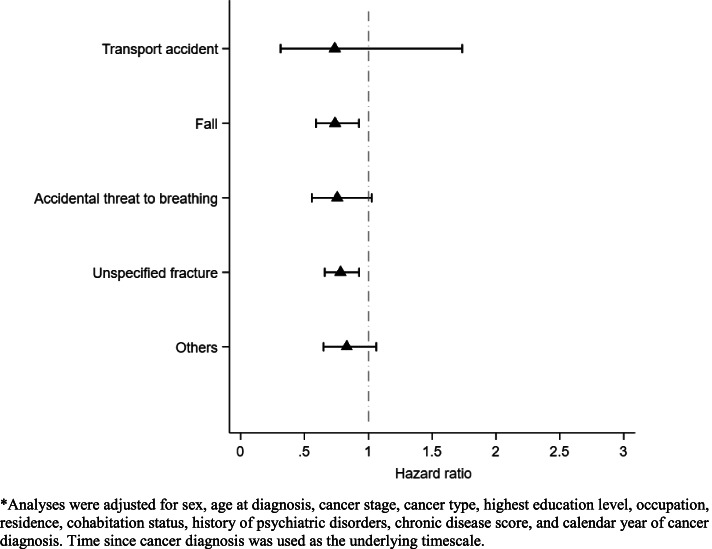
Association of low-dose aspirin use with the risk of death due to different types of accidents, a cohort study of 388,443 cancer patients diagnosed between Oct 2005 and Dec 2014 in Sweden*. *Analyses were adjusted for sex, age at diagnosis, cancer stage, cancer type, highest education level, occupation, residence, cohabitation status, history of psychiatric disorders, chronic disease score, and calendar year of cancer diagnosis. Time since cancer diagnosis was used as the underlying timescale

The risk reduction in death due to accident, in relation to use of low-dose aspirin, was not statistically significant during the first year (HR 0.92; 95% CI: 0.71 to 1.19) but statistically significant thereafter (HR 0.88; 95% CI: 0.78 to 0.98) after cancer diagnosis. The risk reduction was more prominent among male patients, younger patients (< 60), patients with hematopoietic malignancies, patients without a history of psychiatric disorders, patients with a higher Chronic Disease Score, and patients that were retired or unemployed (Supplementary Materials Table S7). In the analysis of causes of death due to accidents, we found a similar reduction in the risks for different types of accidents in relation to use of aspirin (Fig. 2).

## Discussion

Using a nationwide cohort study, we assessed the association between use of low-dose aspirin and non-aspirin NSAIDs and the risk of unnatural deaths among cancer patients. We found that low-dose aspirin use was associated with a 22% lower risk of death due to accident but not suicide whereas use of non-aspirin NSAIDs was not associated with the risk of either death due to accident or suicide. When separately analyzing prevalent use and incident use, we found that prevalent use of either aspirin or non-aspirin NSAIDs was associated with a lower risk of death due to accident, whereas incident use of such medication was not. Our study provides evidence for a potentially beneficial effect of NSAID use, especially low-dose aspirin and long-term use, in reducing severe accidents after cancer diagnosis.

### Strength and limitations

The major strength of this study includes the nationwide population-based cohort design, the complete follow-up, and the prospectively and independently collected information on medication, cancer, and death outcomes, which largely minimized the potential risk of selection and information biases. The use of dispensed rather than prescribed medications, the use of prescription text to define medication status, and the time-varying definition of medication use served to limit potential misclassification of the exposure. Because medications are largely financed by the universal healthcare insurance to each resident in Sweden, the findings of the present study are not likely greatly influenced by other factors such as socioeconomic status.

One limitation of the study is that the Prescribed Drug Register does not include medications administered during hospitalization or in nursing home. This may result in misclassification of the exposure to some extent. However, it is unlikely that such misclassification is differential in terms of the outcomes. Another limitation is that we did not have information on the actual consumption of medications among patients defined as exposed to these medications according to drug dispense. In our study, 84.0% patients ever medicated with low-dose aspirin had at least two dispensing records, indicating that vast majority of the users of low-dose aspirin had likely consumed the dispensed medications. Further, because patients who did not consume medications as expected should ideally have contributed their person time to the off-medication periods rather than on-medication periods, this misclassification is expected to have led to underestimation of the inverse association noted for aspirin use. Because information on other potential confounders such as lifestyle factors was unavailable, and there was a proportion of cancer patients with unknown cancer stage, residual confounding remains a concern. The similar results obtained in the within-individual comparison and after multiple imputation for unknown cancer stage alleviated this concern to some extent. There is also a concern of indication bias, namely that the indications for NSAIDs use - mostly inflammatory conditions - might have biased the estimates of the studied associations. This is likely most relevant for first-time use of aspirin and non-aspirin NSAIDs, for both of which an increased risk of death due to incidents was noted. As a result, the overall association as reported in Table 2 could also have been biased partially due to this. Finally, the possibility of chance findings cannot be excluded completely because of the smaller number of outcomes, and this is especially true for completed suicide.

### Interpretation of results

The excess risks of suicide and accidents among cancer patients have been reported in many countries [4, 6, 32, 33]. Although more than twice as many cancer patients die from accidents than from suicide [6, 31], risk of death due to accident and its underlying reasons has been less investigated. Psychological distress related to receiving a cancer diagnosis and treatment, together with its resultant psychiatric symptoms such as depression, fatigue and poor concentration, may trigger the occurrence of both suicide and accidents in cancer patients [7, 34]. To our knowledge, potential preventive strategies have not been developed to mitigate the elevated risk of these outcomes. In our study, we found that low-dose aspirin use was associated with a lower risk of death due to accident. This finding, in line with previous studies, supports a potential role of aspirin use in reducing stress-related outcomes due to its anti-inflammatory properties [35, 36]. Indeed, both cognitive impairment and peripheral neuropathy have been associated with inflammation in cancer patients, which was further correlated with deficits in motor function [37, 38] and risk of external injuries [39, 40]. Aspirin may intervene in this process and modify the risk. Although not statistically significant, it seems the lowest estimate on risk reduction was observed among patients with severe cancers (cancer in esophagus, liver or pancreas). It is possible that this group of patients can potentially benefit more from the anti-inflammatory property of aspirin due to their high level of either tumor-induced or treatment-associated inflammation [41, 42].

The lack of statistically significant association between low-dose aspirin and risk of death due to suicide may indicate indeed a lack of association. However, it is also possible that an underlying association is masked by methodological issues. This contention is in particular compelling given recent findings showing that aspirin use reduces the risk of depression [21], which is a major predictor of suicide [7]. Unlike unpredictable death due to accident, completed suicide is intentional. During the immediate time period before suicide, patients might stop taking or dispensing medications [43]. This could have led to different kinds of biases. For instance, if the patients had dispensed aspirin recently, they would be counted as exposed to aspirin despite not actually using it. Alternatively, before suicide, patients might be less likely to dispense prescribed medications, leading to an artificial protective effect, i.e., less patients would have dispensed aspirin during the time before suicide compared to expected. Another possible explanation for the lack of a statistically significant association is the lack of statistical power because of the smaller number of completed suicide. Moreover, suicide is known to be a challenge in classifying causes of death, and can be misclassified as deaths due to accident [44]. When combining completed suicide and deaths due to accident as one outcome, we found a 21% lower risk of unnatural deaths associated with the use of low-dose aspirin. A protective effect of low-dose aspirin on the risk of suicide following a cancer diagnosis can therefore not be ruled out.

Prevalent use of non-aspirin NSAIDs, but not incident use, was found to be associated with a lower risk of accidental death. Non-aspirin NSAIDs are a class of drugs with analgesic, anti-inflammatory, and anti-pyretic therapeutic properties [45]. Because of their side effect on gastrointestinal and cardiovascular systems when used chronically [46, 47], non-aspirin NSAIDs are recommended for use at the lowest effective dose, and for short-term use when possible [48]. In our study, the average duration of exposure to non-aspirin NSAIDs (1.4 months, corresponding to 4% of exposed follow-up time) was substantially shorter compared with the average duration of exposure to low-dose aspirin (16 months, corresponding to 77% of exposed follow-up time). This is also reflected by the greater numbers of outcomes among on-medication periods with aspirin compared to the on-medication periods with non-aspirin NSAIDs (see Table 2 for instance). As a result, the null overall association between non-aspirin NSAIDs and risk of death due to accident might be due to chance (or lack of statistical power). In a sensitivity analysis, we observed an inverse association between prevalent use of both aspirin and non-aspirin NSAIDs, but not incident use of either, and risk of death due to accident. These results suggest that long-term use of NSAIDs, whether aspirin or non-aspirin NSAIDs, is likely to be associated with a lower risk of death due to accident among cancer patients. The overall null association between non-aspirin NSAIDs and risk of death due to accident might therefore be attributable to the fact that most of the non-aspirin NSAIDs are used in short term whereas short-term use of such medication is unlikely to be associated with a lower risk of death due to accident among cancer patients. Taken together, these results might indicate the potential role of the anti-inflammatory properties of NSAIDs in modulating the risk of accidents among patients with cancer.

## Conclusions

In conclusion, the use of low-dose aspirin might be associated with a lower risk of death due to accident among patients with cancer.

## Supplementary Information

**Additional file 1.**

## Data Availability

Data from Swedish population and health registers cannot be put into a public data repository due to Swedish law but are available through application to Statistics Sweden (https://www.scb.se/vara-tjanster/bestalla-mikrodata/) and the Swedish National Board of Health and Welfare (https://bestalladata.socialstyrelsen.se/).

## Abbreviations

NSAID: Non-steroidal anti-inflammatory drugs
ICD: International Classifications of Diseases
ATC: Anatomical Therapeutic Chemical
LISA: Longitudinal Integration Database for Health Insurance and Labour Market Studies
HR: Hazard Ratio
CI: Confidence Interval
