# Supplementary material for: NSAID use and unnatural deaths after cancer diagnosis: a nationwide cohort study in Sweden
Source: BMC Cancer. 2022 Jan 17;22:75. doi: 10.1186/s12885-021-09120-9 (PMC8764760; doi:10.1186/s12885-021-09120-9)

**NSAID use and unnatural deaths after cancer diagnosis:**

**a nationwide cohort study in Sweden**

Supplementary Materials

**Table S1. Classification of cancer type.**

| Cancer type | ICD-7 |
| --- | --- |
| Prostate cancer | 177 |
| Breast cancer, female only | 170 |
| Colorectal cancer | 153,154 |
| Non-malignant skin cancer | 191 |
| Lung cancer | 162,163 |
| Lymphatic or hematopoietic cancer | 200-207 |
| Severe cancers (including esophageal, liver and pancreas) | 150,155,156,157 |

**Table S2. Classification of suicide and death due to accident**

| Outcome of interest | ICD-10 |
| --- | --- |
| Death due to suicide | X60-X84 |
| Death due to accident | V01-X59, Y85-Y86 |
| - transport accident | V01-V99 |
| - fall | W00-W19 |
| - accidental threats to breathing | W65-W84 |
| - unspecified fracture | X590 |
| - others | W20-W64, W85-W99, X00-X58, X599, Y85-Y86 |

**Table S3. Association of NSAID use with risk of death due to suicide or accident after cancer diagnosis (with one month after medicated period also counted as exposed period), a cohort study of 388,443 cancer patients diagnosed between Oct 2005 and Dec 2014 in Sweden**

| **Characteristics** | **Completed suicide** | | | **Death due to accident** | | |
| --- | --- | --- | --- | --- | --- | --- |
|  | **N** | **Crude IR (per 1 000 person-years)** | **HR (95% CI)** | **N** | **Crude IR (per 1 000 person-years)** | **HR (95% CI)** |
| **Low-dose aspirin** |  |  |  |  |  |  |
| Off medication (ref) ^a^ | 228 | 0.23 | 1.0 | 1,627 | 1.64 | 1.0 |
| On medication ^b^ | 59 | 0.25 | 0.94 (0.70-1.27) | 651 | 2.73 | 0.88 (0.80-0.97) |
| *As above + cancer stage, cancer type and chronic disease score* | - | - | 1.03 (0.74-1.42) | - | - | 0.79 (0.72-0.87) |
| *As above + history of psychiatric disorders* | - | - | 1.00 (0.73-1.38) | - | - | 0.78 (0.71-0.86) |
| **Non-aspirin NSAIDs** |  |  |  |  |  |  |
| Off medication (ref) ^a^ | 274 | 0.23 | - | 2,219 | 1.86 | - |
| On medication ^b^ | 13 | 0.35 | 1.38 (0.78-2.41) | 59 | 1.58 | 0.97 (0.75-1.26) |
| *As above + cancer stage, cancer type and chronic disease score* | - | - | 1.36 (0.77-2.39) | - | - | 0.93 (0.72-1.21) |
| *As above + history of psychiatric disorders* | - | - | 1.30 (0.74-2.30) | - | - | 0.93 (0.72-1.21) |

Abbreviations: NSAID, non-steroidal anti-inflammatory drugs; IR, incidence rate; HR, hazard ratio; CI, confidence interval.

^a^ Off medication time included follow-up time accumulated among patients without any dispensed NSAIDs during follow-up, as well as the non-medicated periods from patients that had any dispensed NSAIDs.

^b^Analyses were adjusted for sex, age at diagnosis, calendar year of cancer diagnosis, highest educational level, occupation, and cohabitation status; time since cancer diagnosis was used as the underlying timescale.

**Table S4. Association of NSAID use with risk of death due to suicide or accident after cancer diagnosis, comparing on-medication periods to periods without any NSAIDs prescription, a cohort study of 388,443 cancer patients diagnosed between Oct 2005 and Dec 2014 in Sweden**

| **Characteristics** | **Completed suicide** | | | **Death due to accident** | | |
| --- | --- | --- | --- | --- | --- | --- |
|  | N | Crude IR (per 1 000 person-years) | HR (95% CI) | N | Crude IR (per 1 000 person-years) | HR (95% CI) |
| **Low-dose aspirin** |  |  |  |  |  |  |
| Off NSAIDs (ref) ^a^ | 139 | 0.28 | 1.0 | 952 | 1.94 | 1.0 |
| On medication ^b^ | 59 | 0.25 | 0.78 (0.52-1.17) | 651 | 2.80 | 0.81 (0.72-0.91) |
| *As above + cancer stage, cancer type and chronic disease score* | - | - | 0.95 (0.62-1.44) | - | - | 0.75 (0.66-0.86) |
| *As above + history of psychiatric disorders* | - | - | 0.88 (0.58-1.34) | - | - | 0.74 (0.65-0.84) |
| **Non-aspirin NSAIDs** |  |  |  |  |  |  |
| Off NSAIDs (ref) ^a^ | 139 | 0.02 | 1.0 | 952 | 0.16 | 1.0 |
| On medication ^b^ | 13 | 0.03 | 0.78 (0.33-1.83) | 59 | 0.13 | 0.79 (0.58-1.09) |
| *As above + cancer stage, cancer type and chronic disease score* | - | - | 0.85 (0.36-2.05) | - | - | 0.74 (0.54-1.01) |
| *As above + history of psychiatric disorders* | - | - | 0.80 (0.33-1.92) | - | - | 0.74 (0.54-1.01) |

Abbreviations: NSAID, non-steroidal anti-inflammatory drugs; IR, incidence rate; HR, hazard ratio; CI, confidence interval.

^a^ Off NSAIDs included follow-up time accumulated among patients without any dispensed NSAIDs during follow-up.

^b^Analyses were adjusted for sex, age at diagnosis, calendar year of cancer diagnosis, highest educational level, occupation and cohabitation status; time since cancer diagnosis was used as the underlying timescale.

**Table S5. Association of NSAID use with risk of death due to suicide or accident after cancer diagnosis, with additional adjustment for other medications, a cohort study of 388,443 cancer patients diagnosed between Oct 2005 and Dec 2014 in Sweden.**

| **Characteristics** | **Completed suicide** | | | **Death due to accident** | | |
| --- | --- | --- | --- | --- | --- | --- |
|  | **N** | **Crude IR (per 1 000 person-years)** | **HR (95% CI)** | **N** | **Crude IR (per 1 000 person-years)** | **HR (95% CI)** |
| **Low-dose aspirin** |  |  |  |  |  |  |
| Off medication (ref) ^a^ | 228 | 0.23 | 1.0 | 1,627 | 1.63 | 1.0 |
| On medication ^b^ | 59 | 0.25 | 0.96 (0.66-1.39) | 651 | 2.80 | 0.78 (0.70-0.87) |
| *As above + use of opioids* | - | - | 0.96 (0.67-1.40) | - | - | 0.78 (0.70-0.88) |
| *As above + use of anxiolytics* | - | - | 1.04 (0.71-1.51) | - | - | 0.80 (0.72-0.89) |
| *As above + use of antidepressants* | - | - | 1.04 (0.72-1.52) | - | - | 0.80 (0.71-0.89) |
| **Non-aspirin nsaids** |  |  |  | - | - |  |
| Off medication (ref) ^a^ | 274 | 0.02 | 1.0 | 2 219 | 0.15 | 1.0 |
| On medication ^b^ | 13 | 0.03 | 0.95 (0.42-2.18) | 59 | 0.13 | 0.92 (0.68-1.25) |
| *As above + use of opioids* | - | - | 0.95 (0.41-2.17) | - | - | 0.90 (0.67-1.23) |
| *As above + use of anxiolytics* | - | - | 0.98 (0.43-2.24) | - | - | 0.90 (0.67-1.23) |
| *As above + use of antidepressants* | - | - | 0.97 (0.42-2.21) | - | - | 0.91 (0.67-1.23) |

Abbreviations: NSAID, non-steroidal anti-inflammatory drugs; IR, incidence rate; HR, hazard ratio; CI, confidence interval.

^a^ Off medication time included follow-up time accumulated among patients without any dispensed NSAIDs during follow-up, as well as the non-medicated periods from patients that had any dispensed NSAIDs.

^b^Analyses were adjusted for sex, age at diagnosis, calendar year of cancer diagnosis, highest educational level, occupation, cohabitation status, cancer stage, cancer type, Chronic Disease Score and history of psychiatric disorders; time since cancer diagnosis was used as the underlying timescale.

**Table S6. Association of NSAID use with risk of death due to suicide or accident after cancer diagnosis, with multiple imputation for unknown cancer stage, a cohort study of 388,443 cancer patients diagnosed between Oct 2005 and Dec 2014 in Sweden.**

| **Characteristics** | **Completed suicide** | | | **Death due to accident** | | |
| --- | --- | --- | --- | --- | --- | --- |
|  | **N** | **Crude IR (per 1 000 person-years)** | **HR (95% CI)** | **N** | **Crude IR (per 1 000 person-years)** | **HR (95% CI)** |
| **Low-dose aspirin** |  |  |  |  |  |  |
| Off medication (ref) ^a^ | 228 | 0.23 | 1.0 | 1,627 | 1.63 | 1.0 |
| On medication ^b^ | 59 | 0.25 | 0.92 (0.64-1.31) | 651 | 2.80 | 0.88 (0.80-0.98) |
| As above + cancer stage, cancer type and chronic disease score | - | - | 1.00 (0.69-1.46) | - | - | 0.78 (0.70-0.87) |
| As above + history of psychiatric disorders | - | - | 0.96 (0.66-1.38) | - | - | 0.77 (0.69-0.86) |
| **Non-aspirin NSAIDs** |  |  |  |  |  |  |
| Off medication (ref) ^a^ | 274 | 0.02 | 1.0 | 2 219 | 0.15 | 1.0 |
| On medication ^b^ | 13 | 0.03 | 1.02 (0.45-2.32) | 59 | 0.13 | 0.96 (0.71-1.30) |
| As above + cancer stage, cancer type and chronic disease score | - | - | 1.03 (0.45-2.35) | - | - | 0.94 (0.69-1.28) |
| As above + history of psychiatric disorders | - | - | 0.97 (0.42-2.21) | - | - | 0.94 (0.69-1.27) |

Abbreviations: NSAID, non-steroidal anti-inflammatory drugs; IR, incidence rate; HR, hazard ratio; CI, confidence interval.

^a^Off medication time included follow-up time accumulated among patients without any dispensed NSAIDs during follow-up, as well as the non-medicated periods from patients that had any dispensed NSAIDs.

^b^Analyses were adjusted for sex, age at diagnosis, calendar year of cancer diagnosis, highest educational level, occupation and cohabitation status; time since cancer diagnosis was used as the underlying timescale.

**Table S7. Association of aspirin use with risk of death due to accident after cancer diagnosis by patient characteristics, a cohort study of 388,443 cancer patients diagnosed between Oct 2005 and Dec 2014 in Sweden.**

| **Characteristics** | **Death due to accident** | | | | |
| --- | --- | --- | --- | --- | --- |
|  | **Off medication^b^** | | **On medication** | | |
|  | **N** | **Crude IR (per 1 000 person-years)** | **N** | **Crude IR (per 1 000 person-years)** | **HR (95% CI)^a^** |
| **Sex** |  |  |  |  |  |
| men | 960 | 1.91 | 396 | 2.73 | 0.72 (0.62-0.83) |
| women | 667 | 1.33 | 255 | 2.91 | 0.89 (0.75-1.06) |
|  |  |  |  |  |  |
| **Age at diagnosis, years** |  |  |  |  |  |
| <60 | 111 | 0.34 | 9 | 0.47 | 0.44 (0.18-1.12) |
| 60-69 | 249 | 0.74 | 62 | 0.93 | 0.87 (0.62-1.22) |
| 70-79 | 457 | 2.00 | 174 | 2.03 | 0.84 (0.68-1.03) |
| >80 | 810 | 7.37 | 406 | 6.67 | 0.74 (0.65-0.86) |
|  |  |  |  |  |  |
| **Cancer type** |  |  |  |  |  |
| prostate cancer | 299 | 1.30 | 133 | 1.99 | 0.80 (0.63-1.01) |
| breast cancer | 135 | 0.72 | 58 | 2.35 | 0.77 (0.53-1.12) |
| colorectal cancer | 176 | 1.66 | 64 | 2.43 | 0.84 (0.59-1.19) |
| non-melanoma skin cancer | 204 | 3.29 | 112 | 4.83 | 0.77 (0.60-1.00) |
| hematopoietic malignancy | 177 | 2.67 | 59 | 3.45 | 0.63 (0.44-0.89) |
| lung cancer | 136 | 4.88 | 36 | 4.63 | 0.80 (0.48-1.32) |
| severe cancer (esophagus, liver and pancreas) | 79 | 5.72 | 20 | 6.84 | 0.59 (0.27-1.27) |
|  |  |  |  |  |  |
| **Cancer stage at diagnosis^c^** |  |  |  |  |  |
| localized limited | 305 | 0.94 | 151 | 2.36 | 0.85 (0.68-1.07) |
| localized advanced | 112 | 1.83 | 45 | 2.98 | 0.95 (0.62-1.44) |
| regional spread | 168 | 1.74 | 45 | 2.58 | 0.70 (0.47-1.05) |
| distant metastasis | 201 | 5.22 | 71 | 8.97 | 0.99 (0.66-1.50) |
| unknown | 664 | 1.60 | 280 | 2.53 | 0.73 (0.62-0.87) |
| not applicable | 177 | 2.67 | 59 | 3.45 | 0.63 (0.44-0.89) |
|  |  |  |  |  |  |
| **History of psychiatric disorders** |  |  |  |  |  |
| no | 1345 | 1.50 | 525 | 2.62 | 0.75 (0.66-0.85) |
| yes | 282 | 2.63 | 126 | 3.92 | 0.95 (0.74-1.23) |
|  |  |  |  |  |  |
| **Chronic Disease Score** |  |  |  |  |  |
| 0 | 317 | 0.78 | 28 | 1.77 | 1.07 (0.70-1.64) |
| 1-2 | 638 | 1.62 | 162 | 2.70 | 0.88 (0.72-1.07) |
| 3-6 | 589 | 3.24 | 390 | 2.87 | 0.71 (0.62-0.82) |
| >6 | 83 | 4.14 | 71 | 3.46 | 0.69 (0.48-0.98) |
|  |  |  |  |  |  |
| **Educational level** |  |  |  |  |  |
| high | 791 | 1.16 | 276 | 2.19 | 0.73 (0.62-0.86) |
| low | 836 | 2.62 | 375 | 3.53 | 0.82 (0.71-0.95) |
|  |  |  |  |  |  |
| **Cohabitation status** |  |  |  |  |  |
| no | 739 | 1.77 | 309 | 3.12 | 0.80 (0.69-0.92) |
| yes | 550 | 0.96 | 216 | 1.65 | 0.76 (0.64-0.90) |
|  |  |  |  |  |  |
| **Occupation** |  |  |  |  |  |
| employed (blue collar, white collar, farmers, or self-employed) | 85 | 0.24 | 15 | 0.48 | 0.85 (0.44-1.62) |
| retired or unemployed | 1212 | 2.11 | 512 | 2.66 | 0.77 (0.69-0.87) |

Abbreviations: NSAID, non-steroidal anti-inflammatory drugs; IR, incidence rate; HR, hazard ratio; CI, confidence interval.

^a^ Analyses were adjusted for sex, age at diagnosis, cancer stage, cancer type, highest education level, occupation, cohabitation status, history of psychiatric disorder, Chronic Disease Score, and calendar year of cancer diagnosis; time since cancer diagnosis was used as the underlying timescale.

^b^ Off medication time included follow-up time accumulated among patients without any dispensed NSAIDs during follow-up, as well as the non-medicated periods from patients that had any dispensed NSAIDs.

*^c^* Defined by European Network of Cancer Registries Condensed TNM Scheme and International Federation of Gynecology and Obstetrics staging system: localized limited (T-localized/N0/M0 or FIGO 0-I), localized advanced (T-advanced/N0/M0 or FIGO II), regional spread (any T/N+/M0 or FIGO III), distant metastasis (any T/any N/M+ or FIGO IV), or unknown stage. Hematological malignancies were classified as not applicable.

**Figure S1. Illustration of on- and off-medication periods during follow-up using one fictitious patient as an example**

Solid line of axis “time in months” is follow-up time. Dot line of axis “time in months” is the time before follow-up. Patient starts to be at risk from time 0 and onwards. Gray box represents on-medication period, and filled circle stands for occurrence of failure or censoring.


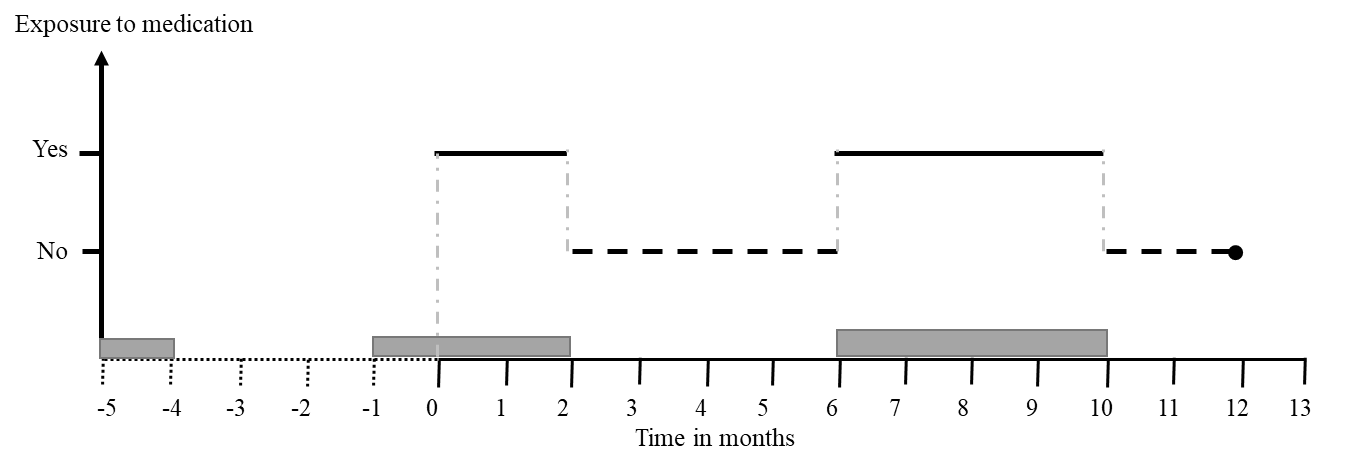


**Figure S2. Cumulative incidence of death due to suicide or accident after cancer diagnosis by years since diagnosis, a cohort study of 388,443 cancer patients diagnosed between Oct 2005 and Dec 2014 in Sweden.**

**A. by use of aspirin**


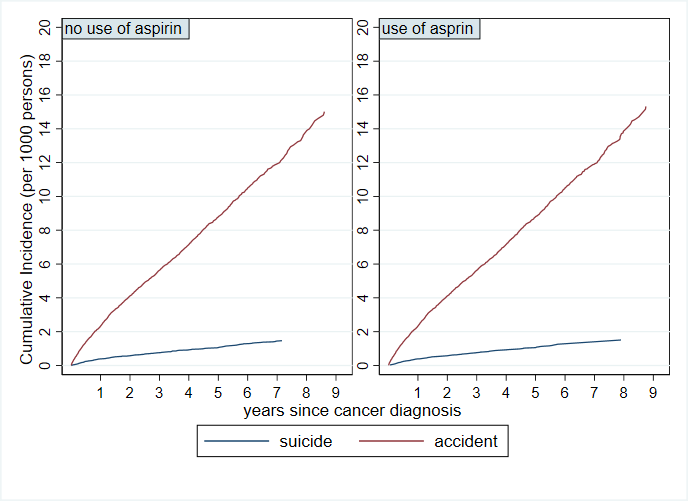


**B. by use of non-aspirin NSAIDs**


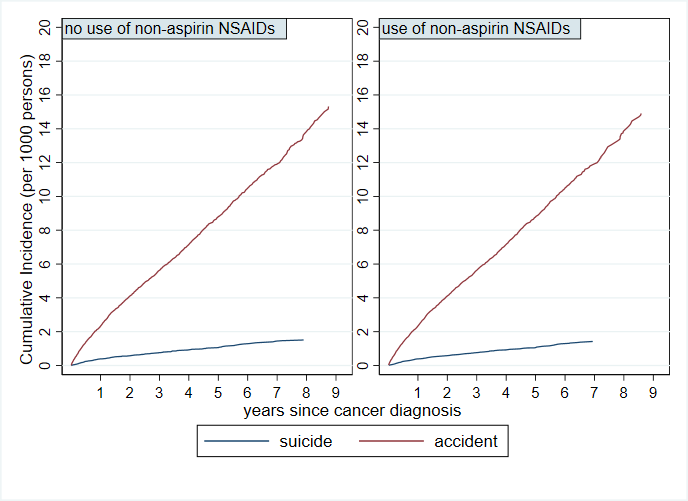

Supplement: Supplementary file 1 — Additional file 1. [file 12885_2021_9120_MOESM1_ESM.docx]
